# NFκB-mediated CXCL1 production in spinal cord astrocytes contributes to the maintenance of bone cancer pain in mice

**DOI:** 10.1186/1742-2094-11-38

**Published:** 2014-03-01

**Authors:** Jie Xu, Ming-Di Zhu, Xin Zhang, Hao Tian, Jin-Hua Zhang, Xiao-Bo Wu, Yong-Jing Gao

**Affiliations:** 1 Pain Research Laboratory, Institute of Nautical Medicine, Jiangsu Key Laboratory of Neuroregeneration, Nantong University, 19 Qixiu Road, Nantong 226001, China; 2 Department of Orthopedics, Affiliated Hospital to Nantong University, Nantong 226001, China; 3 Department of Radiology, Affiliated Hospital to Nantong University, Nantong 226001, China; 4 Department of Urology, Haimen People’s Hospital, Haimen, Jiangsu Province 226100, China

**Keywords:** Bone cancer pain, Chemokines, CXCL1, CXCR2, NFκB, Astrocytes, Astroglia-neuron interaction

## Abstract

**Background:**

Bone cancer pain (BCP) is one of the most disabling factors in patients suffering from primary bone cancer or bone metastases. Recent studies show several chemokines (for example, CCL2, CXCL10) in the spinal cord are involved in the pathogenesis of BCP. Here we investigated whether and how spinal CXCL1 contributes to BCP.

**Methods:**

Mouse prostate tumor cell line, RM-1 cells were intramedullary injected into the femur to induce BCP. The mRNA expression of CXCL1 and CXCR2 was detected by quantitative real-time PCR. The protein expression and distribution of CXCL1, NFκB, and CXCR2 was examined by immunofluorescence staining and western blot. The effect of CXCL1 neutralizing antibody, NFκB antagonist, and CXCR2 antagonist on pain hypersensitivity was checked by behavioral testing.

**Results:**

Intramedullary injection of RM-1 cells into the femur induced cortical bone damage and persistent (>21 days) mechanical allodynia and heat hyperalgesia. Tumor cell inoculation also produced CXCL1 upregulation in activated astrocytes in the spinal cord for more than 21 days. Inhibition of CXCL1 by intrathecal administration of CXCL1 neutralizing antibody at 7 days after inoculation attenuated mechanical allodynia and heat hyperalgesia. In cultured astrocytes, TNF-α induced robust CXCL1 expression, which was dose-dependently decreased by NFκB inhibitor. Furthermore, inoculation induced persistent NFκB phosphorylation in spinal astrocytes. Intrathecal injection of NFκB inhibitor attenuated BCP and reduced CXCL1 increase in the spinal cord. Finally, CXCR2, the primary receptor of CXCL1, was upregulated in dorsal horn neurons after inoculation. Inhibition of CXCR2 by its selective antagonist SB225002 attenuated BCP.

**Conclusion:**

NFκB mediates CXCL1 upregulation in spinal astrocytes in the BCP model. In addition, CXCL1 may be released from astrocytes and act on CXCR2 on neurons in the spinal cord and be involved in the maintenance of BCP. Inhibition of the CXCL1 signaling may provide a new therapy for BCP management.

## Background

Bone cancer pain (BCP) is the most common symptom detected in patients with advanced breast, prostate, and lung cancer [1]. Current treatment strategies often provide inadequate analgesia and unacceptable side effects [2]. Understanding the underlying mechanisms related to the development of BCP is important for effectively treating these patients.

Glial cell-mediated neuroinflammation has been recently shown to play a pivotal role in the pathogenesis of chronic pain [3,4]. Tissue injury/inflammation, nerve injury, and tumor growth can induce glial cells (astrocytes and microglia) to be reactive and release a variety of inflammatory mediators, including proinflammatory cytokines and chemokines, which may augment the nociceptive signals in the spinal cord [5-8]. Chemokines are small secreted proteins and are key molecules involved in the migration and homeostasis of immune cells. Recent studies have shown that some chemokines in the spinal cord are involved in BCP. For example, CCL2 expression is increased in spinal astrocytes and microglia in mice with BCP [9]. Intrathecal administration of CCL2 neutralizing antibody attenuates tumoral hyperalgesia [9,10]. Tumor cell inoculation also induces the increases of CXCL10 and its major receptor CXCR3 in the spinal cord. Blocking the function of CXCL10/CXCR3 pathway via anti-CXCL10 antibody or CXCR3 antagonist prevents the development of BCP and microglial activation [11].

CXCL1 is a member of CXC family and is also known as keratinocyte-derived chemokines (KC) or growth-related oncogene (GRO). CXCL1 is highly expressed in melanoma cell lines and promotes malignant melanoma tumor progression [12]. CXCL1 also modulates neuronal excitability of DRG neurons by increasing sodium currents, potassium currents, and the function of TRPV1 channels [13-15]. In the spinal cord, CXCL1 is upregulated in astrocytes after spinal nerve ligation and contribute to the maintenance of neuropathic pain [16]. However, little is known about whether CXCL1 participates in the maintenance of BCP.

Nuclear factor kappa B (NFκB) is a transcription factor which serves as a transducer between extracellular signals and gene expression. NFκB is involved in CXCL1 transcription in Hs294T malignant melanoma cells [17]. Moreover, emerging evidence indicates that the activation of NFκB following tissue injury or nerve damage is related to the generation of chronic pain [18-20]. Whether NFκB mediates CXCL1 expression in the spinal astrocytes and contributes to BCP needs to be investigated.

In this study, we examined CXCL1 expression and distribution in the spinal cord after inoculation of mouse prostate cell line, RM-1 cells into the femur. We also evaluated the role of NFκB in CXCL1 production and pain hypersensitivity after tumor cell inoculation. As the biological effects of chemokines are mediated via interaction with its G protein-coupled receptor, and CXCR2 is the primary receptor of CXCL1, we further investigated the expression and distribution of CXCR2 in the spinal cord and the antinociceptive effect of CXCR2 antagonist.

## Methods

### Animals and tumor inoculation

Experiments were performed on adult (8 weeks) male C57Bl/6 mice. All mice had free access to food and water with a 12/12 light/dark cycle. All animal procedures in this study were performed according to the guidelines of the International Association for the Study of Pain and approved by the Animal Care and Use Committee of Nantong University. Mice prostate tumor cell line, RM-1, was purchased from the Cell Bank of Type Culture Collection of Chinese Academy of Sciences (Shanghai, China). RM-1 cells were grown in Dulbecco’s modified Eagle medium (DMEM) containing 4,500 mg/L glucose, 100 mg/L penicillin, 100 mg/L streptomycin, and supplemented with 10% fetal bovine serum (FBS) at 37°C. The RM-1 cells were collected following enzymatic digestion, centrifuged, and resuspended in phosphate buffered saline (PBS) in a concentration of 5 × 10^7^ cells/mL. The animals were anesthetized with sodium pentobarbital (50 mg/kg, i.p.). An arthrotomy was done to expose the condyles of the distal femur. PBS containing 10^6^ RM-1 cells (20 μL) was injected into the intramedullary space of the right femur with a 30 G needle and the injection site was sealed with bone wax. Sham control mice were injected with same amount of heat-inactivated RM-1 cells.

### Radiology

To confirm cancer development in the femur, mice were radiographed at 21 days following implantation. The animals were anesthetized with sodium pentobarbital, placed in a prone position, and exposed to X-ray. The images were collected by Digital Diagnost Dual Detector (Philips).

### Drugs and administration

The CXCL1 neutralizing antibody was purchased from Boster (Wuhan, China). SB225002, a potent and selective antagonist of CXCR2, was purchased from Tocris (Bristol, UK). BAY11-7082, a NFκB inhibitor, was purchased from Merck (Merck KGaA, Darmstadt, Germany). Intrathecal injection was made with a 30 G needle between the L5 and L6 intervertebral space to deliver the reagents to the cerebral spinal fluid [21].

### Behavioral analysis

Animals were habituated to the testing environment daily for at least 2 days before baseline testing. The room temperature remained stable for all experiments. For testing mechanical sensitivity, animals were put in boxes on an elevated metal mesh floor and allowed 30 min for habituation before examination. The plantar surface of each hindpaw was stimulated with a series of von Frey hairs with logarithmically incrementing stiffness (0.02-2.56 g, Stoelting, Wood Dale, IL, USA), presented perpendicular to the plantar surface (2 to 3 s for each hair). The 50% paw withdrawal threshold was determined using Dixon’s up-down method [22]. For testing heat sensitivity, animals were put in plastic boxes and allowed 30 min for habituation. Heat sensitivity was tested by radiant heat using Hargreaves apparatus (IITC Life Science Inc., Woodland Hills, CA, USA) and expressed as paw withdrawal latency (PWL). The radiant heat intensity was adjusted so that basal PWL is between 10 and 14 s, with a cutoff of 18 s to prevent tissue damage.

### Primary astrocytes cultures

Primary astrocytes cultures were prepared from cerebral cortexes of neonatal mice (P2) [23]. The cerebral hemispheres were isolated and transferred to ice-cold Hank’s buffer and the meninges were carefully removed. Tissues were then minced into approximately 1 mm pieces, triturated, filtered through a 100 μm nylon screen, and collected by centrifugation at 3,000 g for 5 min. The cell pellets were dispersed with a pipette and resuspended in a medium containing 10% FBS in low glucose DMEM. After trituration, the cells were filtered through a 40 μm screen and then plated into 6-well plates at a density of 2.5 × 10^5^ cells/cm^2^, and cultured for about 10 days. The medium was replaced twice a week with 10% FBS. Dibutyryl cAMP (0.15 mM, Sigma-Aldrich) was added to induce differentiation when the cells were grown to 95% confluence. Prior to stimulation with LPS, OPTI-MEM was replaced. Astrocytes were incubated with TNF-α for different time periods. The treatment of the BAY11-7082 (1, 5, 10 μM) was started 30 min prior to TNF-α treatment. After the treatment, the astrocytes were collected for ELISA or real-time PCR. To check the expression of CXCL1, some cells were cultured onto cover glasses at a density of 2.5 × 10^4^ cells/cm^2^, and fixed by 4% paraformaldehyde for 20 min. Fluorescence double staining of CXCL1 and GFAP were performed (see below).

### Immunohistochemistry and immunocytochemistry

After appropriate survival times, animals were deeply anesthetized with isoflurane and perfused through the ascending aorta with PBS followed by 4% paraformaldehyde with 1.5% picric acid in 0.16 M PB. After the perfusion, the L4-L5 spinal cord segments were removed and postfixed in the same fixative overnight. Spinal cord sections (30 μm, free-floating) were cut in a cryostat and processed for immunofluorescence as we described previously [23]. The sections were first blocked with 2% goat serum for 1 h at room temperature. The sections were then incubated overnight at 4°C with the following primary antibodies: CXCL1 antibody (rabbit, 1:100; Boster), CXCR2 antibody (rabbit, 1:100; Boster), phospho-NF-κB p65 (Ser536) (pNFκB) antibody (mouse, 1:500; Sigma), GFAP antibody (mouse, 1:6,000; Millipore, Billerica, MA, USA), CD11b antibody (Mouse, 1:100, Serotec, Kidlington, UK), NeuN antibody (mouse, 1:3,000, Millipore). The sections were then incubated for 1 h at room temperature with Cy3- or FITC-conjugated secondary antibodies (1:1,000, Jackson ImmunoResearch). For double immunofluorescence, sections were incubated with a mixture of mouse and rabbit primary antibodies followed by a mixture of Cy3- and FITC-conjugated secondary antibodies. The stained sections were examined with a Leica fluorescence microscope, and images were captured with a CCD Spot camera. The specificity of CXCL1 and CXCR2 primary antibodies was tested by preabsorption experiment (Zhang et al. [16]).

For immunocytochemistry, cultured astrocytes were fixed with 4% paraformaldehyde for 20 min and processed for immunofluorescence with CXCL1 (rabbit, 1:100, Boster) and GFAP (mouse, 1:5,000; Millipore) antibody as shown above.

### Real-time PCR

Total RNA was extracted from L4-5 spinal cord with the Trizol reagent (Invitrogen, Carlsbad, CA, USA). One microgram of total RNA was converted into cDNA using PrimeScript RT reagent kit (Takara, Shiga, Japan). The cDNA was amplified using the following primers: CXCL1 forward, 5′-GCT TGA AGG TGT TGC CCT CAG -3′; CXCL1 reverse, 5′-AGA AGC CAG CGT TCA CCA GAC-3′; CXCR2 forward, 5′-TCT GCT CAC AAA CAG CGT CGT A-3′; CXCR2 reverse, 5′-GAG TGG CAT GGG ACA GCA TC-3′; GAPDH forward, 5′-AAA TGG TGA AGG TCG GTG TGA AC-3′; GAPDH reverse, 5′-CAA CAA TCT CCA CTT TGC CAC TG-3′. The SYBR *Premix Ex Taq*™ II kit (Takara) was used for all PCR reactions, which were run on a Rotor-Gene 6000 RT-PCR machine (Hamburg, Germany). The PCR amplifications were performed at 95°C for 30 s, followed by 45 cycles at 95°C for 5 s, 56°C for 30 s, and 72°C for 30 s. The melting curves were performed to validate the utility and specificity of each PCR product. The data were analyzed using Rotor-Gene 6000 series software, and evaluated using the Comparative CT Method (2^-ΔΔCT^).

### ELISA

Mouse CXCL1 ELISA kit was purchased from R&D. Cultured cells were collected after treatment and homogenized in a lysis buffer containing protease and phosphatase inhibitors (Sigma). Protein concentrations were determined by BCA Protein Assay (Pierce, Rockford, IL, USA). For each reaction in a 96-well plate, 100 μg of proteins were used, and ELISA was performed according to manufacturer’s protocol. The standard curve was included in each experiment.

### Western blot

Protein samples were prepared in the same way as for ELISA analysis, and 30 μg of proteins were loaded for each lane and separated on SDS-PAGE gel (10%). After the transfer, the blots were incubated overnight at 4°C with polyclonal antibodies against CXCR2 (1:100, rabbit, Boster) or pNFκB (1:1,000, rabbit, Cell Signaling Technology). For loading control, the blots were probed with GAPDH antibody (1:20,000, mouse, Sigma). These blots were further incubated with HRP-conjugated secondary antibody, developed in ECL solution, and exposed onto film (Millipore) for 1 to 5 min. Specific bands were evaluated by apparent molecular size. The intensity of the selected bands was analyzed using Image J software (NIH, Bethesda, MD, USA).

### Quantification and statistics

The behavioral and real-time PCR data were analyzed by one-way analysis of variance (ANOVA) followed by Newman-Keuls *post hoc* test. For western blot, the density of specific bands was measured with Image J. CXCR2 and p-NFκB levels were normalized to loading control (GAPDH) [24]. For the analysis of CXCL1- or GFAP-immunoreactivity, four to five sections from the L4-L5 spinal cord segments were randomly selected. An image in a square on the medial two-thirds of the superficial dorsal horn (laminae I–III) was captured under × 20 objective [25]. A numerical value of the immunofluorescence intensity was calculated with Image J (NIH). The intensity of the background was subtracted in each section and the CXCL1 or GFAP intensity was expressed as fold increase compared to control [24]. All data were expressed as mean ± SEM. Differences between two groups were compared using Student’s t-test. The criterion for statistical significance was *P* <0.05.

## Results

### Intramedullary inoculation of RM-1 cells produces the destruction of cortical bone and bone cancer pain

**Figure 1:**
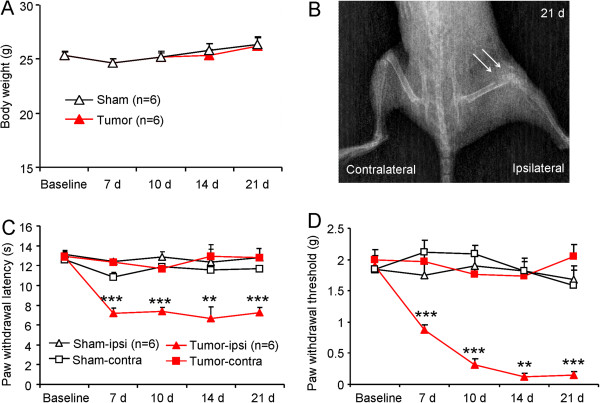
**RM-1 cell inoculation induces BCP. (A)** The animals’ body weight was increased in 21 days in both sham-control and tumor-inoculated animals. **(B)** Radiography shows cortical bone damage in the distal one-third of the right femur (arrows) at 21 days after inoculation. **(C, D)** Behavioral tests show that mice displayed both heat hyperalgesia and mechanical allodynia in the ipsilateral paw after RM-1 cell inoculation. The PWL decreased at 7 days and maintained for more than 21 days **(C)**. The PWT progressively decreased from 7 days to 14 days and maintained at 21 days **(D)**. **P <0.01, ***P <0.001 *vs*. sham-ipsi. One-way ANOVA followed by Newman-Keuls test. n = 6 mice per group.

After RM-1 prostate tumor cells were inoculated into the intramedullary space of mouse femur, the overall conditions of mice were good and the body weight was gradually increased in 3 weeks (Figure 1A). By day 21 after inoculation, the loss of medullary bone and destruction of cortical bone were clearly observed in the distal one-third of the right femur (Figure 1B). No radiological change was found in the contralateral femur (Figure 1B) or control animals treated with heat-inactivated tumor cells.

Pain behavioral studies showed that tumor cell inoculation produced an obvious pain hypersensitivity, which was characterized by heat hyperalgesia (increased response to a noxious heat stimulus) and mechanical allodynia (painful response to a normally innocuous mechanical stimulus) in the right hindpaws of inoculated mice. For heat sensitivity, the paw withdrawal latency (PWL) of inoculated mice to heat stimulation was decreased from 12.8 ± 0.4 s before inoculation to 7.2 ± 0.5 s on day 7 (*P* <0.001), and maintained on day 10 (7.4 ± 0.4 s, *P* <0.001), day 14 (6.7 ± 1.1 s, *P* <0.01), and day 21 (7.2 ± 0.6 s, *P* <0.001) (Figure 1C), indicating the development of heat hyperalgesia. For mechanical sensitivity, the paw withdrawal threshold (PWT) of the ipsilateral paw, in response to von Frey hair stimulation, was decreased from 1.9 ± 0.16 g before inoculation to 0.9 ± 0.09 g on day 7 (*P* <0.001), 0.3 ± 0.10 g on day 10 *(P* <0.001), 0.12 ± 0.05 g on day 14 (*P* <0.01), and 0.15 ± 0.05 g on day 21 (*P* <0.001, Figure 1D), indicating the progressive development of mechanical allodynia. The contralateral paw of inoculated mice or bilateral paws of sham-treated mice did not show changes in pain sensitivity (Figure 1C,D).

### CXCL1 is persistently increased in spinal cord astrocytes after RM-1 cell inoculation

**Figure 2:**
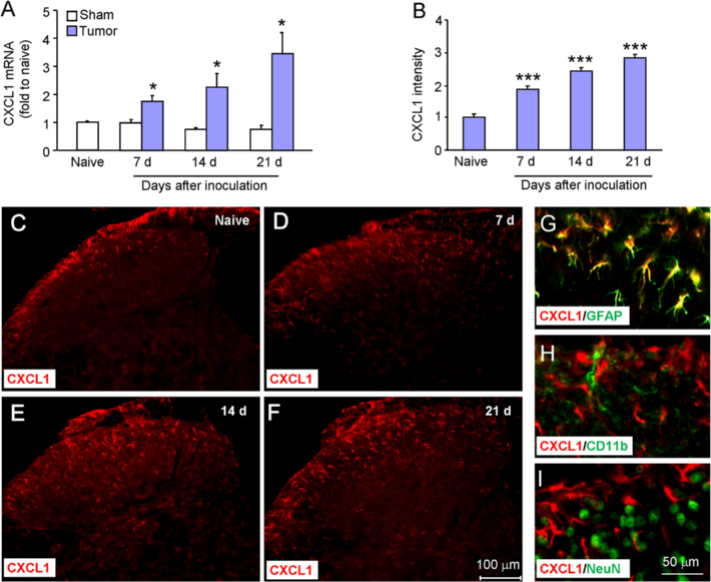
**RM-1 cell inoculation induces CXCL1 upregulation in spinal astrocytes. (A)** Real-time PCR results show the increase of CXCL1 mRNA expression in the spinal cord after inoculation. CXCL1 mRNA upregulation was gradually increased from 7 days to 21 days. *P <0.05 *vs*. sham control. n = 4 mice per group. **(B-F)** Immunostaining shows the CXCL1-IR was increased in the spinal cord at 7 days **(D)**, 14 days **(E)**, and 21 days **(F)**. ***P <0.001 *vs*. naive. n = 4 mice per group. **(G-I)** Double staining shows CXCL1 was colocalized with astrocytic marker, GFAP **(G)**, but not with microglial marker CD11b **(H)** or neuronal marker NeuN **(I)**.

To examine CXCL1 expression in the spinal cord, we first performed quantitative real-time PCR. As shown in Figure 2A, CXCL1 mRNA expression was not changed in sham animals, but significantly increased at 7 days (*P* <0.05), 14 days (*P* <0.05), and 21 days (*P* <0.05) in inoculated animals. We then checked CXCL1 protein expression by immunostaining. Tumor cell inoculation induced a marked increase of CXCL1 expression in the ipsilateral spinal cord at 7 days, 14 days, and 21 days (Figure 2B-D). The statistical analysis of CXCL1-immunoreactive (IR) intensity showed a gradual increase from 7 days to 21 days after tumor cell inoculation (*P* <0.001, Figure 2B).

To define the cellular distribution of CXCL1, we performed double staining of CXCL1 with different cell markers. The results showed that CXCL1-IR was colocalized with the astrocytic marker GFAP (Figure 2G), but not with microglial marker CD11b (Figure 2H) or neuronal marker NeuN (Figure 2I), indicating the expression of CXCL1 by astrocytes.

### Inhibition of CXCL1 by neutralizing antibody attenuates RM-1 cell inoculation-induced pain hypersensitivity

**Figure 3:**
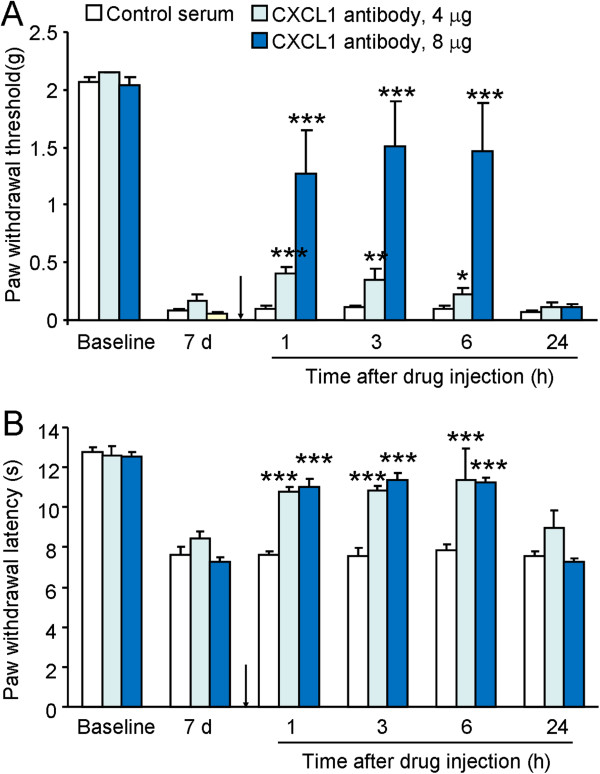
**Intrathecal injection of CXCL1 neutralizing antibody attenuates bone cancer pain.** CXCL1 neutralizing antibody at a lower dose (4 μg) had mild effect on RM-1 cell inoculation-induced pain hypersensitivity **(A, B)**, whereas the neutralizing antibody at a higher dose (8 μg) reversed inoculation-induced mechanical allodynia **(A)** and heat hyperalgesia **(B)** for more than 6 h. *P <0.05, **P <0.01, ***P <0.001 *vs*. control serum. n = 6 mice per group.

To investigate the role of endogenous CXCL1 in BCP, we intrathecally injected a CXCL1 neutralizing antibody at 7 days after inoculation and checked pain behaviors. CXCL1 neutralizing antibody at the dose of 4 μg partly attenuated mechanical allodynia at 1 h (*P* <0.001), 3 h (*P* <0.01), and 6 h (*P* <0.05). High dose (8 μg) of CXCL1 neutralizing antibody almost reversed mechanical allodynia for 6 h (Figure 3A). Meanwhile, CXCL1 neutralizing antibody at doses of 4 μg and 8 μg attenuated heat hyperalgesia at 1 h, 3 h and 6 h (*P* <0.001, Figure 3B). These data suggest CXCL1 is involved in tumor cell inoculation-induced pain hypersensitivity.

### Astrocytes activation in the spinal cord after RM-1 cell inoculation

**Figure 4:**
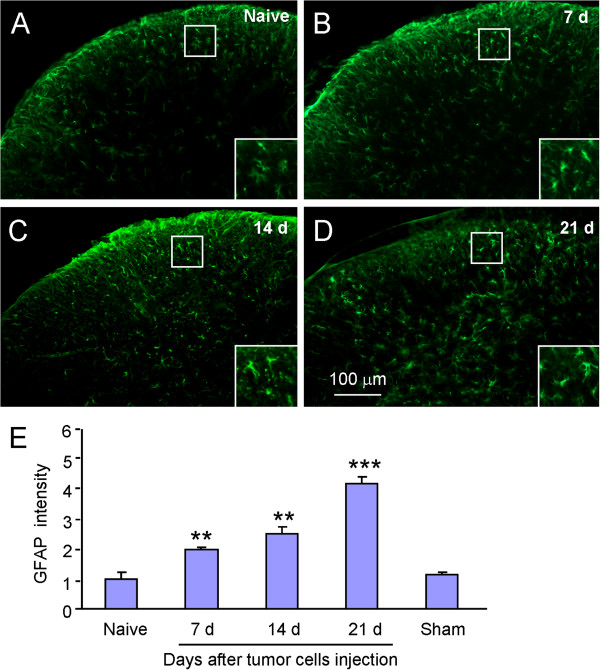
**RM-1 cell inoculation induces GFAP upregulation in the spinal cord. (A-D)** GFAP-IR was mild in naïve mice **(A)**, but increased at 7 days **(B)**, 14 days **(C)**, and 21 days **(D)** in inoculated mice. **(E)** Statistical analysis shows increased GFAP intensity after RM-1 cell inoculation. **P <0.01, ***P <0.001 *vs*. sham. n = 4 mice per group.

Because tumor cell inoculation induced CXCL1 increase in spinal astrocytes, we then examined astrocytes activation by checking GFAP expression in the spinal cord. In naïve animals, GFAP-positive astrocytes appeared to be in a resting state (Figure 4A). At 7 days after tumor cell inoculation, the astrocyte profiles appeared larger and had more processes compared to naïve (Figure 4A,B). Intense astrocytic responses were discernible on day 14 (Figure 4C) and day 21 (Figure 4D). The statistical analysis of GFAP-IR intensity showed a gradual increase of GFAP expression in 21 days after tumor cell inoculation. GFAP expression was not increased in sham animals (Figure 4E).

### TNF-α induces CXCL1 upregulation via activation of NFκB in cultured astrocytes

**Figure 5:**
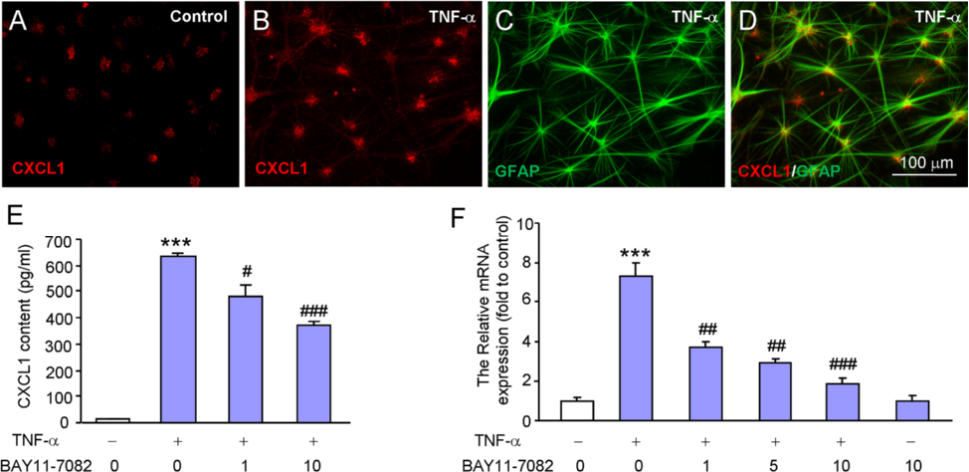
**TNF-α induces NFκB-dependent CXCL1 increase in cultured astrocytes. (A-D)** CXCL1 was expressed in control astrocytes **(A)** and increased at 1 h after TNF-α incubation **(B)**. Double staining of CXCL1 **(B)** with GFAP **(C)** shows the expression of CXCL1 by astrocytes **(D)**. **(E)** ELISA results show TNF-α-induced CXCL1 upregulation was decreased by pretreatment with NFκB inhibitor, BAY11-7082. ***P <0.001 *vs*. control. #*P* <0.05, ###*P* <0.001 *vs*. TNF-α treatment. **(F)** Quantitative PCR shows TNF-α-induced CXCL1 mRNA increase was decreased by BAY11-7082. ***P <0.001 *vs*. control. ##*P* <0.01, ###*P* <0.001 *vs.* TNF-α treatment.

NFκB is a transcriptional factor and has been demonstrated to regulate the transcription of many inflammatory mediators, including those for chemokines and proinflammatory cytokines [20,26]. To check if NFκB is involved in CXCL1 production in astrocytes, we first prepared primary astrocyte cultures from cerebral cortexes of neonatal mice (P2) and stimulated with TNF-α. As shown in Figure 5A, TNF-α incubation for 1 h increased CXCL1-IR (Figure 5A,B). Double staining with GFAP showed that majority of CXCL1-IR was colocalized with astrocytes (Figure 5C,D). We then examined the effects of NFκB inhibitor, BAY11-7082 on CXCL1 expression by ELISA and RT-PCR. Pretreatment of BAY11-7082, 30 min before TNF-α treatment, decreased CXCL1 protein expression by 24.4% and 40.9% at the doses of 1 μM and 10 μM, respectively (Figure 5E). In addition, BAY11-7082 decreased CXCL1 mRNA expression by 50.0%, 60.7%, and 74.2% at the doses of 1 μM, 5 μM, and 10 μM, respectively (Figure 5F). These data suggest NFκB is critical for mediating TNF-α-induced CXCL1 production in cultured astrocytes.

### NFκB activation in spinal astrocytes after RM-1 cell inoculation

**Figure 6:**
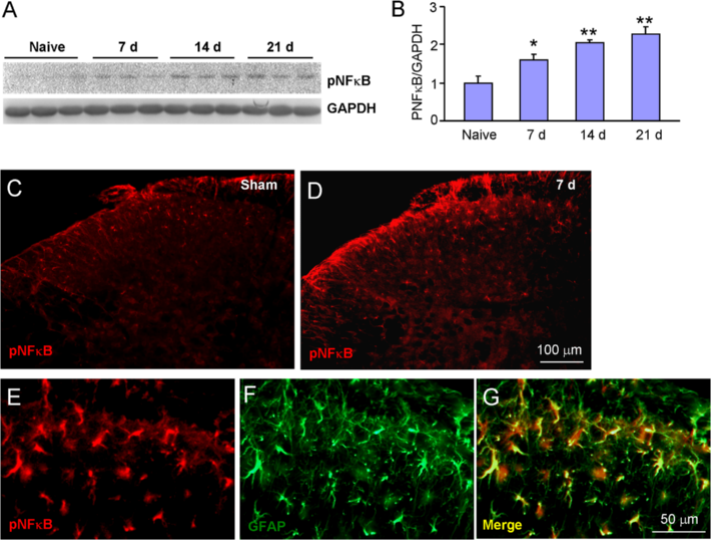
**RM-1 cell inoculation increases pNFκB expression in spinal astrocytes. (A,B)** Western blot shows that tumor cell inoculation increased pNFκB expression in the spinal cord at 7 days, 14 days, and 21 days. *P <0.05, **P <0.01 *vs*. naive. One-way ANOVA followed by Newman-Keuls test. n = 4 mice per group. **(C, D)** Immunostaining shows that pNFκB was expressed in the superficial dorsal horn in sham animals **(C)**, increased at 7 days in inoculated animals **(D)**. **(E-G)** Double staining showed pNFκB was colocalized with GFAP.

To check whether NFκB-CXCL1 pathway would be involved in tumor cell inoculation-induced CXCL1 upregulation and pain hypersensitivity, we first checked NFκB activation in the spinal cord after tumor cell inoculation. Western blot showed phosphorylated NFκB (pNFκB) expression was gradually increased from 7 days to 21 days (Figure 6A,B). Immunostaining further showed a low expression of pNFκB in sham-treated mice (Figure 6C) and an increased expression in inoculated mice (Figure 6D). Double staining showed pNFκB was predominantly colocalized with GFAP (Figure 6E-G).

### Inhibition of NFκB attenuated bone cancer pain and decreased CXCL1 upregulation in the spinal cord

**Figure 7:**
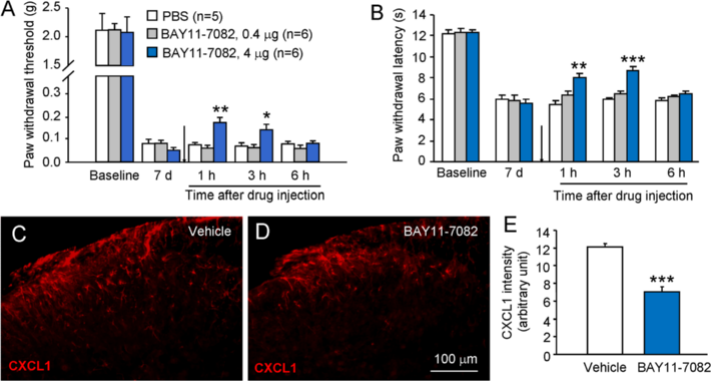
**NFκB inhibitor attenuated RM-1 cell inoculation-induced pain hypersensitivity and upregulation of CXCL1 in the spinal cord. (A, B)** Intrathecal injection of NFκB inhibitor, BAY11-7082 at the dose of 0.4 μg had no effect on mechanical allodynia or heat hyperalgesia, whereas at the dose of 4 μg attenuated mechanical allodynia **(A)** and heat hyperalgesia **(B)** at 1 h and 3 h. *P <0.05, **P <0.01, ***P <0.001 *vs.* vehicle. One-way ANOVA followed by Newman-Keuls test. **(C, D)** Immunostaining of CXCL1 in the spinal cord in vehicle and BAY11-7082-treated animals. **(E)** The CXCL1-IF intensity was decreased by BAY11-7082. ***P <0.001 *vs*. vehicle. n = 4 mice per group.

We then check the role of NFκB in the maintenance of BCP. We intrathecally injected BAY11-7082 (0.4 μg and 4 μg) at 7 days after inoculation. BAY11-7082 at the dose of 0.4 μg had no effect on mechanical allodynia, but 4 μg of this compound significantly attenuated mechanical allodynia at 1 h (*P* <0.01) and 3 h (*P* <0.05, Figure 7A). The high dose of BAY11-7082 also attenuated heat hyperalgesia at 1 h (*P* <0.01) and 3 h (*P* <0.001, Figure 7B). BAY11-7082 also significantly decreased CXCL1-IR in the spinal cord (P <0.001, Figure 7C-E). These data suggest that NFκB is involved in the tumor cell inoculation-induced CXCL1 upregulation in spinal astrocytes and pain hypersensitivity.

### CXCR2 is persistently upregulated in spinal neurons and involved in bone cancer pain

**Figure 8:**
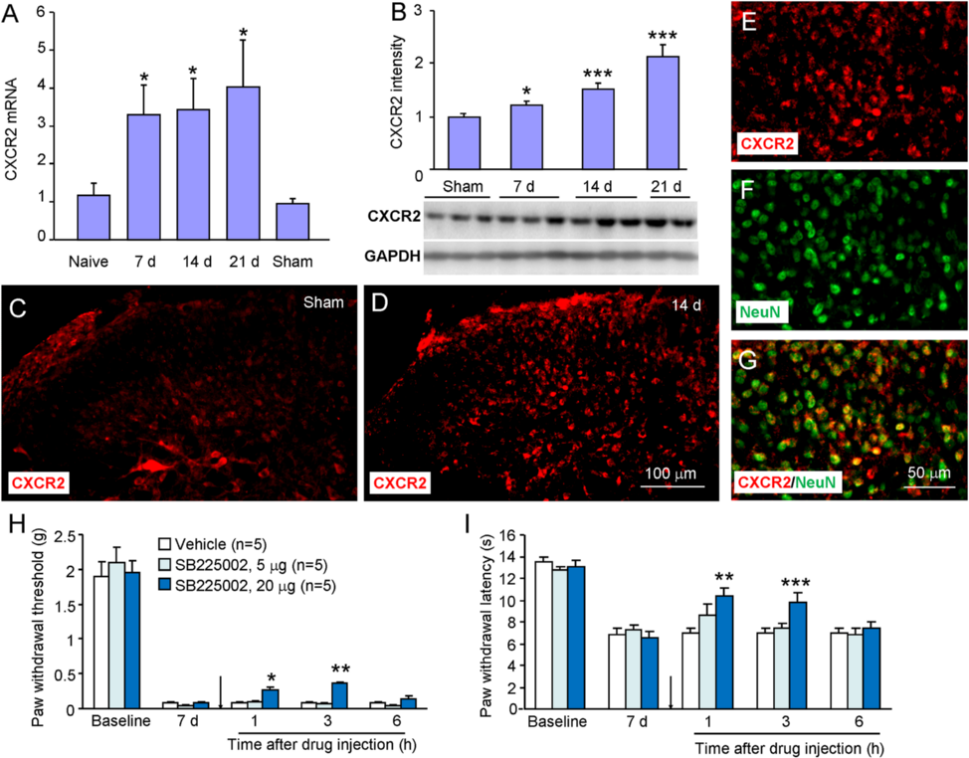
**RM-1 cell inoculation increases CXCR2 mRNA and protein expression in the spinal cord. (A)** Real-time PCR results show the increase of CXCR2 mRNA expression in the spinal cord. CXCR2 mRNA was increased from 7 days to 21 days after inoculation. *P <0.05 *vs*. sham. n = 4 mice per group. **(B)** Western blot shows time course of CXCR2 protein expression in the spinal cord after inoculation **(B)**. n = 3 mice per group. **(C, D)** Immunostaining shows the CXCR2 expression in the spinal cord in sham **(C)** and inoculated **(D)** animals. CXCR2-IR was increased at 7 days after inoculation. **(E-G)** Double staining shows CXCR2 was colocalized with neuronal marker NeuN. **H**, **I**. SB2205002 attenuated RM-1 cell inoculation-induced mechanical allodynia **(H)** and heat hyperalgesia **(I)**. *P <0.05; **P <0.01; ***P <0.001 *vs*. vehicle.

CXCR2 is the major receptor of CXCL1. We further investigated CXCR2 expression and distribution in the spinal cord after tumor cell inoculation. RT-PCR showed CXCR2 mRNA was increased at 7 days and maintained for more than 21 days (*P* <0.05, Figure 8A). Western blot showed CXCR2 expression was gradually increased from 7 days to 21 days (Figure 8B). Immunostaining further showed a low expression of CXCR2 in naïve mice (Figure 8C) and an increased expression in inoculated mice (Figure 8D). Double staining showed that majority CXCR2-IR was colocalized with NeuN, indicating the predominant production of CXCR2 by spinal neurons (Figure 8E-G).

To investigate the role of CXCR2 in the BCP, a selective and potent CXCR2 antagonist, SB225002 (5 μg and 20 μg) was intrathecally injected at 7 days after tumor cell inoculation. SB225002 at the dose of 5 μg had no effect on mechanical allodynia or heat hyperalgesia, but SB225002 at the dose of 20 μg significantly attenuated mechanical allodynia and heat hyperalgesia at 1 h and 3 h (Figure 8H,I), suggesting the involvement of CXCR2 in BCP.

## Discussion

To investigate the mechanisms involved in the pathogenesis of BCP, animal models have been developed by injecting tumor cells lines (for example, osterolytic 2472 sarcoma, B16 melanoma, Walker 256 mammary gland carcinoma) into bones (for example, femur or tibia) [27]. Studies have shown that different cell line has unique phenotype in the extent of bone destruction, the type and severity of pain behaviors [27,28]. Here, we, for the first time injected mouse prostate cell line, RM-1 cells into mice femur. This cell line induced obvious cortical bone destruction of the femur and severe and persistent mechanical allodynia and heat hyperalgesia, suggesting it is suitable for BCP model. Using this model, our study demonstrated first that CXCL1 was dramatically increased in activated astrocytes in the spinal cord after tumor cell inoculation. Inhibition of CXCL1 attenuated inoculation-induced pain hypersensitivity. Second, NF-κB was involved in CXCL1 production in cultured astrocytes and was activated in spinal astrocytes after inoculation. Inhibition of NFκB not only alleviated BCP but also decreased CXCL1 upregulation in the spinal cord. Third, CXCR2, the major receptor of CXCL1 was increased in spinal cord neurons. Intrathecal injection of CXCR2 antagonist attenuated BCP. These data suggest that NFκB/CXCL1 and CXCR2 play an important role in the maintenance of BCP via astroglial-neuronal interaction in the spinal cord.

### CXCL1 upregulation in activated astrocytes in the spinal cord and the involvement in bone cancer pain

Recent studies have shown that CXCL1 expression in the spinal cord is changed in different pain models. Contusion injury of spinal cord induces rapid but transient (6 h) CXCL1 mRNA in the spinal cord [29]. Spinal nerve ligation induces persistent CXCL1 mRNA and protein increase, which was peaked at 10 days, declined at 21 days [16]. In this study, CXCL1 mRNA and protein was progressively increased in 21 days after tumor cell inoculation, indicating CXCL1 play distinct roles in different pain conditions.

Accumulating evidence supports that glial cells (astrocytes and microglia) are activated in the spinal cord after tumor cells inoculation in the skin or bone marrow [30-35]. Consistent with these studies, we found that the expression of astrocytic marker, GFAP was increased from 7 days to 21 days in the spinal cord. Spinal microglia was also activated for more than 21 days in this model (unpublished observation). In addition, the glial activation was correlated with the pain behavior. It has been shown that intrathecal injection of glial function inhibitor, fluorocitrate or microglia inhibitor, minocycline attenuated allodynia induced by Walker 256 inoculation [33,36,37], supporting the view that spinal astrocytes and microglia are involved in the pathogenesis of BCP.

It is increasingly recognized that astrocytes mediate neuroinflammation by the release of gliotransmitters such as proinflammatory cytokines (for example, IL-1β) [30,38] and chemokines (for example, CCL2 and CCL7) [23,39]. Our present study showed that CXCL1 was produced by spinal astrocytes after tumor cells inoculation. In agreement with present results, CXCL1 mRNA is found to be upregulated in spinal astrocytes after spinal cord injury in rats [40] or spinal nerve ligation in mice [16]. CXCL1 is induced in brain astrocytes by neuronal injury and intracerebroventricular administration of endothelin-1 [41,42]. CXCL1 is also selectively expressed in hypertropic astrocytes after active multiple sclerosis lesions in humans [43,44]. These data suggest that CXCL1 is one of astrogliotransmitters that regulate neuroinflammation.

Several chemokines, such as CCL2, CX3CL1, CCL5, or CXCL10 are recently found to be involved in BCP [10,11,45,46]. In our study, intrathecal injection of CXCL1 neutralizing antibody at 7 days after inoculation attenuated tumor cell inoculation-induced mechanical allodynia and heat hyperalgesia. This antibody also attenuated spinal nerve ligation-induced neuropathic pain at 10 days, but not at 1 day after ligation [16], indicating the major role of CXCL1 in the maintenance of chronic pain.

### NFκB mediates CXCL1 upregulation and contributes to bone cancer pain

The proinflammatory cytokine TNF-α is an essential trigger for the development of neuropathic pain [16,47]. TNF-α mRNA is rapidly (1 d) and dramatically (five-fold) increased in the spinal cord after spinal nerve ligation [16]. TNF-α protein is upregulated in the spinal cord at 3 days after tumor cell inoculation [48]. In addition, combined deletion of TNF receptor (TNFR) 1 and TNFR2 inhibits fibrosarcoma inoculation-induced astrogliosis in the spinal cord and mechanical allodynia [35]. These data indicate that TNF-α can be rapidly increased in the spinal cord and acts on astrocytes to initiate astrocytes activation and BCP. In cultured astrocytes, TNF-α induces rapid and dramatic CXCL1 upregulation [16]. Intrathecal injection TNF-α not only increases CXCL1 expression in spinal astrocytes but also induces heat hyperalgesia [16]. These data suggest that the activation of NFκB/CXCL1 signaling after tumor cell inoculation may also be triggered by TNF-α increase.

NFκB regulates the transcription of many inflammatory mediators, including those for chemokines, proinflammatory cytokines, and adhesion molecules [20,26]. NFκB is found to regulate CXCL1 transcription in Hs294T malignant melanoma cells [17]. Here, NFκB mediated CXCL1 production in cultured astrocytes and spinal astrocytes. In addition, inoculation persistently increased NFκB activation in the spinal cord. Evidence suggests that the activation of NFκB following tissue injury or nerve damage is related to the development and maintenance of neuropathic pain. For example, inhibition of NFκB activation in the spinal level by specific inhibitors or lentivirus partly prevents the development of neuropathic pain [20,26]. NFκB inhibitor also attenuates established neuropathic pain [20,49]. Our results showed that intrathecal NFκB inhibitor at 7 days after inoculation attenuated tumoral hypersensitivity and decreased CXCL1 level, supporting the role of NFκB in the maintenance of BCP via CXCL1 production in the spinal cord.

Our data further showed that NFκB was predominantly expressed in astrocytes of the spinal cord. Consistently, pNFκB expression in astrocytes was found in the spinal cord following spinal nerve injury [24] and in the medullary dorsal horn following the inferior alveolar nerve injury [49]. However, activated NFκB is also found in macrophages/microglia in the spinal cord after spinal cord injury [50] or spinal nerve injury in rats [51]. The discrepency of the cellular distribution of NFκB in the spinal cord may be due to different animal species or different antibodies, which need to be further clarified in the future.

### CXCL1/CXCR2 signaling mediates astroglial-neuronal interaction in bone cancer pain

Chemokines act through a family of seven transmembrane G protein-coupled receptors to exert their biological effects. CXCR2 is the major receptor of CXCL1 [52,53]. The CXCR2 receptor has been detected on neurons [54,55], oligodendrocyte progenitors [44,56], and microglia [57,58] in brain. In DRG, CXCR2 are expressed in neurons and CXCL1 increases the sodium currents, potassium currents in small diameter rat sensory neurons [13,14]. In this study, tumor cell inoculation increased CXCR2 expression in spinal neurons. Inhibition of CXCR2 by its selective antagonist attenuated tumoral hypersensitivity, suggesting the involvement of neuronal CXCR2 in BCP.

Glial-neuronal interaction has been implicated to contribute to central sensitization under pathological conditions [5]. Here the respective expression of CXCL1 and CXCR2 in astrocytes and neurons suggest they may be involved in astroglial-neuronal interaction. Our recent data showed that intrathecal injection of CXCL1 induced rapid, CXCR2-dependent ERK and CREB activation mainly in spinal cord neurons. It is known that the activated ERK can be translocated into nucleus, activates transcription factors including CREB, and further regulates gene transcription (for example, c-Fos and COX-2) to maintain central sensitization and chronic pain [59]. Therefore, CXCL1 may regulate BCP through upregulation of pain-related proteins. In addition, we observed that CXCL1 increases NMDA-induced currents on Lamina II neurons of the spinal cord (unpublished observation), suggesting CXCL1 may also be involved in the maintenance of central sensitization through direct regulation of neuronal excitability.

## Conclusions

In this study, we found that inoculation of RM-1 cells into mouse femur induced bone destruction and pain hypersensitivity. In association with these changes, chemokine CXCL1 and its receptor CXCR2 were, respectively, increased in spinal astrocytes and neurons. Moreover, NFκB was involved in the production of CXCL1 in spinal astrocytes. Our data suggest that CXCL1/CXCR2-mediated astroglial-neuronal interaction contributes to the maintenance of tumoral hypersensitivity. CXCL1 signaling may serve as a novel target for the treatment of metastatic prostate cancer-induced BCP.

## Abbreviations

BCP: Bone cancer pain; CXCL1: Chemokine CXC motif ligand 1; CXCR2: Chemokine CXC motif receptor 2; GAPDH: Glyceraldehyde-3-phophate dehydrogenase; GFAP: Glial fibrillary acidic protein; NFκB: Nuclear factor kappa B; PWL: Paw withdrawal latency; PWT: Paw withdrawal threshold; TNF-α: Tumor necrosis factor-α.

## Competing interests

The authors declare no competing interests.

## Authors’ contributions

JX carried out the animal surgery, behavioral testing, cell culture, immunohistochemistry, and western blot experiments. MDZ and XZ participated in the animal surgery and behavioral testing. HT did the X-ray scan. JHZ and XBW participated to the design of the experiments. YJG conceived of the project, coordinated and supervised the experiments, analyzed data, and wrote the manuscript. All authors read and approved the final manuscript.
